# Theory Before the Test: How to Build High-Verisimilitude Explanatory Theories in Psychological Science

**DOI:** 10.1177/1745691620970604

**Published:** 2021-01-06

**Authors:** Iris van Rooij, Giosuè Baggio

**Affiliations:** 1 Donders Institute for Brain, Cognition and Behaviour, Radboud University; 2 Department of Language and Literature, Norwegian University of Science and Technology

**Keywords:** theory development, formal modeling, computational analysis, psychological explanation, levels of explanation, computational-level theory, theoretical cycle

## Abstract

Drawing on the philosophy of psychological explanation, we suggest that psychological science, by focusing on effects, may lose sight of its primary explananda: psychological capacities. We revisit Marr’s levels-of-analysis framework, which has been remarkably productive and useful for cognitive psychological explanation. We discuss ways in which Marr’s framework may be extended to other areas of psychology, such as social, developmental, and evolutionary psychology, bringing new benefits to these fields. We then show how theoretical analyses can endow a theory with minimal plausibility even before contact with empirical data: We call this the *theoretical cycle*. Finally, we explain how our proposal may contribute to addressing critical issues in psychological science, including how to leverage effects to understand capacities better.

A substantial proportion of research effort in experimental psychology isn’t expended directly in the explanation business; it is expended in the business of discovering and confirming effects.

—Robert Cummins (2000, p. 120)

Psychological science has a preoccupation with “effects.” However, effects are explananda (things to be explained), not explanations. The Stroop effect, for instance, does not explain why naming the color of the word “red” written in green takes longer than naming the color of a green patch. That just *is* the Stroop effect.^1^ The effect itself is in need of explanation. Moreover, effects such as we experimentally test in the laboratory are secondary explananda for psychology. Ideally, we do not construct theories *just* to explain effects.^2^ Rather, the Stroop effect, the McGurk effect, the primacy and recency effects, and visual illusions, for example, serve to arbitrate between competing explanations of the capacities for cognitive control, speech perception, memory, and vision, respectively.

Primary explananda are key phenomena defining a field of study. They are derived from observations that span far beyond, and often even precede, the testing of effects in the lab. Cognitive psychology’s primary explananda are the cognitive capacities that humans and other animals possess. These capacities include, in addition to those already mentioned, those for learning, language, perception, concept formation, decision-making, planning, problem-solving, reasoning, and so on.^3^ Only in the manner in which we postulate that such capacities are exercised do our explanations of capacities come to imply effects. An example is given by Cummins (2000):

Consider two multipliers, M1 and M2. M1 uses the standard partial products algorithm. . . . M2 uses successive addition. Both systems have the capacity to multiply. . . . But M2 also exhibits the “linearity effect”: computation is, roughly, a linear function of the size of the multiplier. It takes twice as long to compute 24 × *N* as it does to compute 12 × *N*. M1 does not exhibit the linearity effect. Its complexity profile is, roughly, a step function of the number of digits in the multiplier. (p. 123)

This example illustrates two points. First, many of the effects studied in our labs are by-products of how capacities are exercised. They may be used to test different explanations of how a system works: For example, by giving a person different pairs of numerals and by measuring response times, one can test whether the person’s timing profile fits M1 or M2, or any different M′. Second, candidate explanations of capacities (multiplication) come in the form of different algorithms (e.g., partial products or repeated addition) computing a particular function (i.e., the product of two numbers). Such algorithms are not devised to explain effects; rather, they are posited as a priori candidate procedures for realizing the target capacity.

Although effects are usually discovered empirically through intricate experiments, capacities (primary explananda) do not need to be discovered in the same way (Cummins, 2000). Just as we knew that apples fall straight from the trees (rather than move upward or sideways) before we had an explanation in terms of Newton’s theory of gravity,^4^ so too do we already know that humans can learn languages, interpret complex visual and social scenes, and navigate dynamic, uncertain, culturally complex social worlds. These capacities are so complex to explain computationally or mechanistically that we do not know yet how to emulate them in artificial systems at human levels of sophistication. The priority should be the discovery not of experimentally constructed effects but of plausible explanations of real-world capacities. Such explanations may then provide a theoretical vantage point from which to also explain known effects (secondary explananda) and perhaps to guide inquiry into the discovery of new informative ones.

This approach is not the one psychological science has been pursuing in recent decades; nor is it what the contemporary methodological-reform movement in psychological science has been recommending. Methodological reform so far seems to follow the tradition of focusing on establishing statistical effects, and, arguably, the reform has even been entrenching this bias. The reform movement has aimed primarily at improving methods for determining which statistical effects are replicable (cf. debates on preregistration; Nosek et al., 2019; Szollosi et al., 2020), and there has been relatively little concern for improving methods for generating and formalizing scientific explanations (for notable exceptions, see Guest & Martin, 2021; Muthukrishna & Henrich, 2019; Smaldino, 2019; van Rooij, 2019). But if we are already “overwhelmed with things to explain, and somewhat underwhelmed by things to explain them with” (Cummins, 2000, p. 120), why do psychological scientists expend so much energy hunting for more and more effects? We see two reasons besides tradition and habit.

One is that psychological scientists may believe in the need to build large collections of robust, replicable, uncontested effects before even thinking about starting to build theories. The hope is that, by collecting many reliable effects, the empirical foundations are laid on which to build theories of mind and behavior. As reasonable as this seems, without a prior theoretical framework to guide the way, collected effects are unlikely to add up and contribute to the growth of knowledge (Anderson, 1990; Cummins, 2000; Newell, 1973). An analogy may serve to bring the point home. In a sense, trying to build theories on collections of effects is much like trying to write novels by collecting sentences from randomly generated letter strings. Indeed, each novel ultimately consists of strings of letters, and theories should ultimately be compatible with effects. Still, the majority of the (infinitely possible) effects are irrelevant for the aims of theory building, just as the majority of (infinitely possible) sentences are irrelevant for writing a novel.^5^ Moreover, many of the *relevant* effects (sentences) may never be discovered by chance, given the vast space of possibilities.^6^ How can we know which effects are relevant and informative and which ones are not? To answer this question, we first need to build candidate theories and determine which effects they imply.

Another reason, not incompatible with the first, may be that psychological scientists are unsure how to even start to construct theories if those theories are not somehow based on effects. After all, building theories of capacities is a daunting task. The space of possible theories is, prima facie, at least as large as the space of effects: For any finite set of (naturalistic or controlled) observations about capacities, there exist (in principle) infinitely many theories consistent with those observations. However, we argue that theories may be built by following a *constructive strategy* and meeting key *plausibility constraints* to rule out from the start theories that are least likely to be true: We refer to this as the *theoretical cycle*. An added benefit of this cycle is that theories constructed in this way already have (minimal) verisimilitude *before* their predictions are tested: This may increase the likelihood that confirmed predicted effects turn out to be replicable (Bird, 2018). The assumptions that have to be added to theories to meet those plausibility constraints provide means for (a) making more rigorous tests of theory possible and (b) restricting the number and types of theories considered for testing, channeling empirical research toward testing effects that are most likely to be relevant (more on this later).

This article aims to make accessible ideas for doing exactly this. We present an approach for building theories of capacities that draws on a framework that has been highly successful for this purpose in cognitive science: Marr’s levels of analysis.

## What Are Theories of Capacities?

A capacity is a dispositional property of a system at one of its levels of organization: For example, single neurons have capacities (firing, exciting, inhibiting) and so do minds and brains (vision, learning, reasoning) and groups of people (coordination, competition, polarization). A capacity is a more or less reliable ability (or disposition or tendency) to transform some initial state (or “input”) into a resulting state (“output”).

Marr (1982/2010) proposed that, to explain a system’s capacities, we should answer three kinds of questions: (a) What is the nature of the function defining the capacity (the input-output mapping)? (b) What is the process by which the function is computed (the algorithms computing or approximating the mapping)? (c) How is that process physically realized (e.g., the machinery running the algorithms)? Marr called these computational-level theory, algorithmic-level theory, and implementational-level theory, respectively. Marr’s scheme has occasionally been criticized (e.g., McClamrock, 1991) and variously adjusted (e.g., Anderson, 1990; Griffiths et al., 2015; Horgan & Tienson, 1996; Newell, 1982; Poggio, 2012; Pylyshyn, 1984), but its gist has been widely adopted in cognitive science and cognitive neuroscience, where it has supported critical analysis of research practices and theory building (see Baggio et al., 2012a, 2015, 2016; Isaac et al., 2014; Krakauer et al., 2017). We also see much untapped potential for it in areas of psychology outside of cognitive science.

Following Marr’s views, we propose the adoption of a top-down strategy for building theories of capacities, starting at the computational level. A top-down or function-first approach (Griffiths et al., 2010) has several benefits. First, a function-first approach is useful if the goal is to “reverse engineer” a system (Dennett, 1994; Zednik & Jäkel, 2014, 2016). As Marr stated, “an algorithm is . . . understood more readily by understanding the nature of the problem being solved than by examining the mechanism . . . in which it is embodied” (Marr, 1982/2010, p. 27; see also Marr, 1977).

Knowing a functional target (“what” a system does) may facilitate the generation of algorithmic- and implementational-level hypotheses (i.e., how the system “works” computing that function). Reconsider, for instance, the multiplication example from above: By first expressing the function characterizing the capacity to multiply (*f*(*x,y*) = *xy*), one can devise different algorithms realizing this computation (M1 or M2). If there is no functional target it is difficult or impossible to come up with ways of computing that target. This relates to a second benefit of a function-first approach: It allows one to assess candidate algorithmic or implementational theories for whether they indeed compute or implement that capacity as formalized (Blokpoel, 2018; Cummins, 2000). A third benefit, beyond cognitive psychology, is that social, developmental, or evolutionary psychologists may be more interested in using theories of capacities as explanations of patterns of behavior of agents or groups over time, *in the world*, rather than in the internal mechanisms of those capacities, say, *in the brain or mind*, which is more the realm of cognitive science and cognitive neuroscience.

Psychological theories of capacities should generally be (a) mathematically specified and (b) independent of the details of implementation. The strategy is to try to precisely produce theories of capacities meeting these two requirements, unless evidence is available that this is impossible, for example, that the capacity cannot be modeled in terms of functions mapping inputs to outputs (Gigerenzer, 2020; Marr, 1977). A computational-level theory of a capacity is a specification of input states, *I*, and output states, *O*, and the theorized mapping, *f: I* → *O*. For the multiplication example, the input would be the set of pairs of numbers (ℕ × ℕ), the range would be the set of numbers (ℕ), and the function *f*: ℕ × ℕ → ℕ would be defined such that *f*(*a,b*) = *ab*, for each *a, b* ∈ ℕ. The mapping *f* need not be numerical. It can also be qualitative, structural, or logical. For instance, a computational-level theory of coherence-based belief updating could specify the input as a network *N* = (*P, C*) of possible beliefs (modeled by a set of propositions *P*), in which beliefs in the network may cohere or incohere with each other (modeled by positive or negative connections *C* in the network), a set of currently held beliefs (modeled as truth assignment *T: P* → {*believed to be true, believed to be false*}), and new information that contradicts, conflicts, or is otherwise incoherent with one or more of the held beliefs, *D*. The output could be a belief revision (modeled as a new truth assignment *T*′) that maintains internal coherence as much as possible while accommodating new information, that is, *f*(*N, T, D*) = *T*′ (for applications in the domain of moral, social, legal, practical, and emotional judgments and decision-making, see Thagard, 2000, 2006; Thagard & Verbeurgt, 1998).

Marr’s computational-level theory has often been applied to information-processing capacities as studied by cognitive psychologists. However, Marr’s framework can be extended beyond its traditional domains. First, to the extent that *cognitive* capacities also figure in explanations in other subfields of psychology, Marr’s framework naturally extends to these domains. A few areas in which the approach has been fruitfully pursued include (a) social cognition, for instance, in social categorization (Klapper et al., 2018), mentalizing or “theory of mind” (Baker et al., 2009; Michael & MacLeod, 2018; Mitchell, 2006; Thagard & Kunda, 1998), causal attribution (De Houwer & Moors, 2015), moral cognition (Mikhail, 2008), signaling and communication (Frank & Goodman, 2012; Moreno & Baggio, 2015), and social attachment (Chumbley & Steinhoff, 2019); (b) cognitive development, for instance, in theory of mind (Goodman et al., 2006), probabilistic and causal learning (Bonawitz et al., 2014; Gopnik & Bonawitz, 2015), self-directed learning (Gureckis & Markant, 2012), pragmatic communication (Bohn & Frank, 2019), analogical processing (Gentner, 1983, 2010), and concept formation (Carey, 2009; Kinzler & Spelke, 2007); and (c) cognitive evolution, for instance, in cognitive structures and architectures that aim to account for language, social cognition, reasoning and decision-making (Barrett, 2005; Carruthers, 2006; Cosmides & Tooby, 1995; Fodor, 2000; Lieder & Griffiths, 2020; Marcus, 2006).

Second, and this is a less conventional and less explored idea, the framework can also be applied to noncognitive or nonindividual capacities of relevance to social, developmental, and evolutionary psychology and more. Preliminary explorations into computational-level analyses of noncognitive or nonindividual capacities can be found in work by Krafft and Griffiths (2018) on distributed social processes, Huskey et al. (2020) on communication processes, Rich et al. (2020) on natural and/or cultural-evolution processes, and van Rooij (2012) on self-organized processes.

Later in this article we spell out our approach to theory building using examples. To encourage readers to envisage applications of the approach to their own domains of expertise and to more complex phenomena than those we can cover here, we provide a stepwise account of what is involved in constructing theories of psychological capacities in general. Following Marr’s (1982/2010) successful cash register example, we foresee that more abstract illustrations demonstrating general principles can encourage a wider and more creative adoption of these ideas.

## First Steps: Building Theories of Capacities

We have proposed that theories of capacities may be formulated at Marr’s computational level. A computational-level theory specifies a capacity as a property-satisfying computation *f*. This idea applies across domains in psychology and for capacities at different levels of organization. How does one build a computational-level theory *f* of some capacity *c*? Or better yet, how does one build a *good* computational-level theory?

A first thought may be to derive *f* from observations of the input-output behavior of a system having the capacity under study. However, for anything but trivial capacities, where we can exhaustively observe (or sample) the full input domain,^7^ this is unlikely to work. The reason is that computational-level theories (or *any* substantive theories) are grossly underdetermined by data. The problem that we cannot deduce (or even straightforwardly induce) theories from data is a limitation, or perhaps an attribute, of all empirical science (the Duhem-Quine thesis; Meehl, 1997; Stam, 1992). Still, one may *abduce* hypotheses, including computational-level analyses of psychological capacities. Abduction is reasoning from observations (not limited to experimental data; more below) to possible explanations (Haig, 2018; Niiniluoto, 1999; P. Thagard, 1981; P. R. Thagard, 1978). It consists of two steps: generating candidate hypotheses (abduction proper) and selecting the “best” explanatory one (inference to the best explanation, or IBE). Note, however, that IBE is only as good as the quality of the candidates: The best hypothesis might not be any good if the set does not contain any good hypotheses (Blokpoel et al., 2018; Kuipers, 2000; van Fraassen, 1985). For this reason it is worth building a set of good candidate theories *before* selecting from the set.

Abduction is sensitive to background knowledge. We cannot determine which hypotheses are good (verisimilar) by considering only locally obtained data (e.g., data for a toy scenario in a laboratory task). We should interpret any data in the context of our larger “web of beliefs,” which may contain anything we know or believe about the world, including scientific or commonsense knowledge. One does not posit a function *f* in a vacuum. What we already know or believe about the world may be used to create a first rough set of candidate hypotheses for *f* for any and all capacities of interest. One can either cast the net wide to capture intuitive phenomena and refine and formalize the idea in a well-defined *f* (Blokpoel, 2018; van Rooij, 2008) or, alternatively, make a first guess and then adjust it gradually on the basis of the constraints that one later imposes: The first sketch of an *f* need not be the final one; what matters is how the initial *f* is constrained and refined and how the rectification process can actually drive the theory forward. Theory building is a creative process involving a dialectic of divergent and convergent thinking, informal and formal thinking.

What are the first steps in the process of theory building? Theorists often start with an initial intuitive verbal theory (e.g., that decisions are based on maximizing utilities, that people tend toward internally coherent beliefs, that meaning in language has a systematic structure). The concepts used in this informal theory should then be formally defined (e.g., utilities are numbers, beliefs are propositions, and meanings of linguistic expressions can be formalized in terms of functions and arguments; numbers, propositions, functions, and arguments are all well-defined mathematical concepts). The aim of formalization is to cast initial ideas using mathematical expressions (again, of any kind, not just quantitative) so that one ends up with a well-defined function *f*—or at least a *sketch* of *f*. Once this is achieved, follow-up questions can be asked: Does *f* capture one’s initial intuitions? Is *f* well defined (no informal notions are left undefined)? Does *f* have all the requisite properties and no undesirable properties (e.g., inconsistencies)? If inconsistencies are uncovered between intuitions and formalization, theorists must ask themselves if they are to change their intuitions, the formalization, or both (van Rooij et al., 2019, Chapter 1; for a tutorial, see van Rooij & Blokpoel, 2020). In practice, it always takes several iterations to arrive at a complete, unambiguously formalized *f* given the initial sketch.

Let us illustrate the first steps of theory construction with an example—*compositionality*, a property of the meanings that can be expressed through language. Speakers of a language know intuitively that the meaning of a phrase, sentence, or discourse is codetermined by the meanings of its constituents: For example, “broken glass” means what it does by virtue of the meanings of “broken” and “glass,” plus the fact that composing an adjective (“broken”) and a noun (“glass”) in that order is licensed by the rules of English syntax. Compositionality is the notion that people can interpret the meaning of a complex linguistic expression (a sentence, etc.) as a function of the meanings of its constituents and of the way those constituents are syntactically combined (Partee, 1995). It is the task of a computational theory of syntax and semantics to formalize this intuition. Like other properties of the outputs of psychological capacities, compositionality comes in degrees (Box 1): A system has that capacity just in case it can produce outputs (meanings) showing a higher-than-chance degree of compositionality but not necessarily perfect compositionality.

**Box 1. Possible Objections**

One could object that computational-level theorizing is possible only for *cognitive* (sub)systems, whereas other types of systems require fundamentally different ways of theorizing. We understand this objection in two ways: First, there is something special about the biophysical realization of cognition that makes Marr’s computational level apply only to it (e.g., that brains are computational in ways other systems are not); and second, computational-level analyses intrinsically assume that capacities have functional purposes or serve goals, whereas noncognitive systems (e.g., evolution or emergent group processes) do not.

To counter the first objection, we note that *multiple realizability* (Chalmers, 2011; Dennett, 1995; Miłkowski, 2016) is the bedrock of Marr’s approach: Any function *f* can be physically realized in different systems, even at different levels of organization. Consider the capacity for *sorting*. Its inputs are items varying with respect to a quantity that can be ordered (e.g., the unordered list of numbers 89254631) and gives as output the items in order of magnitude (the ordered list 12345689). This ordering capacity may be physically implemented in several ways. One individual could perform the ordering, a computer program could do it, or a group of people could implement the capacity in a distributed way. In the last of these cases, each individual need not have access to the input array or need not even be aware that they are partaking in a collective behavior that is *ordering* (see figure in this box for an illustration).

**Figure:**
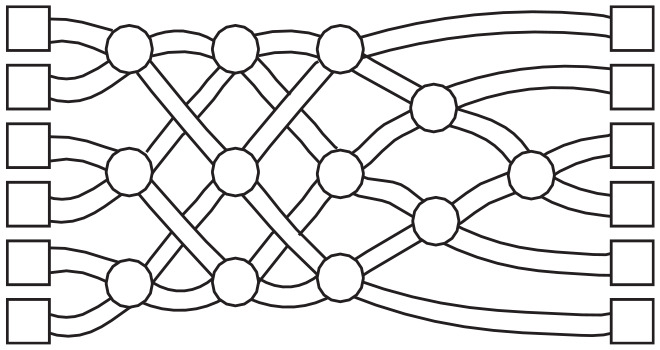
A sorting network. Imagine a maze structured this way; each of six people, walking from left to right, enters a square on the left. Every time two people meet at any node (circle) they compare their height. The shorter of the two turns left next, and the taller turns right. At the end of the maze, people end up sorted by height. This holds regardless of which six people enter the maze and of what order they enter the maze. Hence, the maze (combined with the subcapacity of people for making pairwise comparisons) has the capacity for sorting people by height. Adapted from https://www.csunplugged.org, under a CC BY-SA 4.0 license.

We note that a system may produce outputs in which the target property comes only in degrees: For example, the network in the figure in this box may not always produce a perfect sorting by height if the people entering the maze do not meet at every node; even then, (a) the outputs tend to show a greater degree of ordering than is expected by chance, and (b) under relevant idealizations (i.e., people meet at every node) the system can still produce a complete, correct ordering: Together this illustrates the system’s *sorting capacity*. The fact that target properties come in degrees holds generally; see our discussion of compositionality in the text.

Functions, so conceived, may describe capacities at any level of organization: We see no reason to reserve computational-level explanations only to levels of organization traditionally considered in cognitive science. Even within cognitive psychology, the computational level may be (and has been) applied at different levels of organization—from various neural levels (e.g., feature detection) to persons or groups (e.g., communication). A Marr-style analysis applies regardless of the level of organization at which the primary explananda are situated; hence, it need not be limited to the domain of cognitive psychology.

To counter the second objection, we note that computational-level theories are usually considered to be normative (e.g., rational or optimal, in a realist or instrumentalist “as if” sense; Chater & Oaksford, 2000; Chater et al., 2003; Danks, 2008, 2013; van Rooij et al., 2018), but that is not formally required. A computational-level analysis is a mathematical object, a function *f*, mapping some input domain to an output domain (Egan, 2010, 2017). Any normative interpretation of an *f* is just an add on—an additional, independent level of analysis (Danks, 2008; van Rooij et al., 2018). Marr did suggest that the theory “contains separate arguments about *what* is computed and *why*” (1982, p. 23), but the meaning of “why” has been altered over time by (especially, Bayesian) modelers, as requiring that computational theories are idealized optimization functions serving rational goals (Anderson, 1990, 1991; Chater & Oaksford, 1999). Such an explanatory strategy may have heuristic value for abducing computational-level theories (see text), but it is mistaken to see this strategy as a necessary feature of Marr’s scheme.

Compositionality is a useful example in this context because it holds across cognitive and noncognitive domains and has important social, cultural, and evolutionary ramifications (Table 1), as may be expected from a core property of the human-language capacity. Compositionality is therefore used here to illustrate the applicability of Marr’s framework across areas of cognitive, developmental, social, cultural, and evolutionary psychology. For example, cognitive psychologists may be interested in explaining a person’s capacity to assign compositional meaning to a given linguistic expression, like a vision scientist may be interested in explaining how perceptual representations of visual objects arise from representations of features or parts (the “binding problem”; Table 1, row 1). In all cases covered in Table 1, a “sketch” of a computational theory can be provided as a first step in theory building. A sketch requires that the capacity of interest, the explanandum, is identified with sufficient precision to allow the specification of the inputs, or initial states, and the outputs, or resulting states, of the function *f* to be characterized in full detail in the theoretical cycle. At this stage, we need not say much about the *f* itself, the algorithms that compute it, and the physical systems that implement the algorithms. Moreover, the sketch need not assume anything about the goals (if any) that the capacity serves in each case (Box 1). A discussion of the goals of compositionality, for example, would rather require that a sketch is in place. Are compositional languages easier to learn or to use (Kirby et al., 2008; Nowak & Baggio, 2016)? Is compositional processing one computational resource among others, harnessed only in particular circumstances (Baggio, 2018; Baggio et al., 2012b)? These questions about compositionality’s goals are easier to address when a sketch of *f* is in place. In general, questions about the goals and purposes of the capacity need not affect how either *f* or the output property are defined (Table 1; Box 1).

**Table 1.** Sketches of Computational-Level Analyses of Explananda Involving Compositionality in Different Domains of Psychological Science

Psychological domain	Example explanandum (compositionality)	Computational-level theory (sketch) *f*	Example explananda from other subdomains
Cognitive	The capacity to assign a compositional meaning to a linguistic expression	*Input*: Complex linguistic expression *u_1_, . . ., u_n_*, with elementary parts *u_i_* Output: Meaning of input μ(u_1_, . . ., u_n_), such that μ(u_1_, . . ., u_n_) = **c**(μ(u_1_), . . ., μ(u_n_)), where **c** is a composition operation	The capacity to recognize complex perceptual objects with parts (binding problem)
Development	The capacity to develop comprehension and production skills for a compositional language	*Input*: Basic sensorimotor and cognitive capacities (e.g., memory, precursors of theory of mind), a linguistic environment *Output*: A cognitive capacity *f*_c_ for processing compositional language	The capacity to develop, e.g., fine motor control, abstract arithmetic and geometric skills, etc.
Learning	The capacity to learn a (second or additional) compositional language	*Input*: Basic sensorimotor and cognitive capacities; a linguistic environment; a cognitive capacity *f*_c_ for compositional language understanding and production *Output*: A new cognitive capacity *f*_c_′ that is also compositional	The capacity to learn a new motor skill related to one already mastered, e.g., from ice skating to skiing (skill transfer)
Biological evolution	The capacity to evolve comprehension and production skills for a compositional language	*Input*: A capacity for assigning natural or conventional meanings to signals *Output*: A cognitive capacity *f*_c_ for compositional language use	The capacity to evolve, e.g., fine motor control, spatial representation, navigation, etc.
Social interaction; cultural evolution	The capacity of groups and populations to jointly create new compositional communication codes	*Input*: An arbitrary assignment of meanings to strings *Output*: A compositional assignment of meanings to strings	The capacity of groups or populations to jointly create structured norms and rituals (“culture”); division of labor

## Further Steps: Assessing Theories in the Theoretical Cycle

**Fig. 1.**
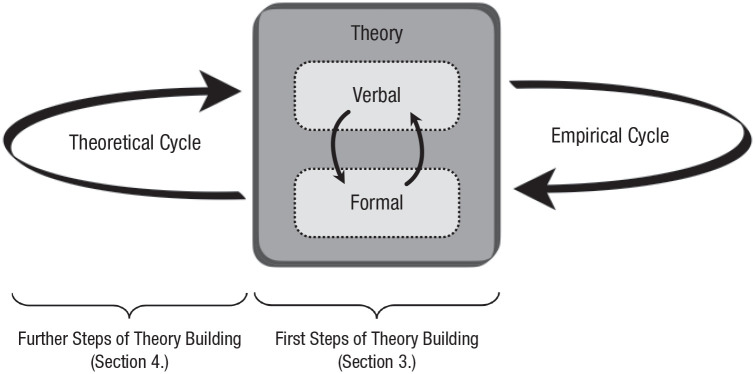
The empirical cycle is familiar to most psychological scientists: The received view is that our science progresses by postulating explanatory hypotheses, empirically testing their predictions (including, but not limited to, effects), and revising and refining the hypotheses in the process. Explanatory hypotheses often remain verbal in psychological research. The first steps of (formal) theory building include making such verbal theories formally explicit. In the process of‘ formalization the verbal theory may be revised and refined. Theory building does not need to proceed with empirical testing right away. Instead, theories can be subjected to rigorous theoretical tests in what we refer to as the theoretical cycle. This theoretical cycle is aimed at endowing the (revised) theory with greater a priori plausibility (verisimilitude) before assessing the theory’s empirical adequacy in the empirical cycle.

Once an initial characterization of *f* is in place, one must ask follow-up questions that probe the *verisimilitude* of *f*. This leads to a crucial series of steps in theory development that are often overlooked in psychological theorizing. Even if one’s intuitive ideas are on the right track and *f* is formalized and internally consistent, it might still lack verisimilitude. A traditional way of testing a theory’s verisimilitude is by deriving predictions from *f* and investigating whether they are borne out when put to an empirical test. Using empirical tests to update and fine-tune a theory is the modus operandi of the *empirical cycle*. We argue that even before (and interlaced with) putting computational-level theories to empirical tests, they can be put to *theoretical tests*, in what we call the *theoretical cycle* (Fig. 1), in which one assesses whether one’s formalization of intuitive, verbal theories satisfies certain theoretical constraints on a priori plausibility.

The hypothetical^8^ example below from the domain of action planning appears simple, but as we demonstrate later it is actually quite complex. One can think of an organism foraging as engaging in *ordering a set of sites to visit*, starting and ending at its “home base,” such that the ordering has overall satisfactory value (e.g., the total cost of travel to the sites in that particular order yields a good trade-off between energy expended for travel and amount of food retrieved). This intuitive capacity can be formalized as follows^9^:

Foraging *f*

*Input*: A set of sites *S* = {*s*_0_, *s*_1_, *s*_2_, . . ., *s_n_*}, each site *s*_i_ ∈ *S* with *i* > 0, hosts a particular amount of food *g*(*s*) ∈ ℕ, and for each pair of sites *s_i_, s_j_* ∈ *S*, there is a cost of travel, *c*(*s_i_, s_j_*) ∈ ℕ.

*Output*: An ordering π(*S*) = [*s*^0^, *s*^1^, . . ., *s*^n^, *s*^0^] of the elements in *S* such that *s*^0^ = *s*_0_ and the sum of foods collected at *s*^1^, . . ., *s*^n^ exceeds the total cost of the travel, that is,

$$\sum_{s\in S}\;g(s)\;\geq \;c(s^{n},\;s^{0})\;+\;\sum_{s^{i},\;s^{i\;+\;1}\;\in \;\pi (S)}c(s^{i},\;s^{i+1})$$

Some arbitrary choices were made here that might matter for the theory’s explanatory value or verisimilitude. For example, we could have formalized the notion of “good trade-off” by defining it as (a) maximizing the amount of food collected given an upper bound on the cost of travel, (b) minimizing the amount of travel given a lower bound on the amount of food collected, or (c) maximizing the difference between the total amount of food collected and the cost of travel. We could also have added free parameters, weighing differentially the importance of maximizing the amount of food and of minimizing the cost of travel.

In the theoretical cycle, one explores the assumptions and consequences of a given set of formalization choices, thereby assessing whether a computational-level theory is making unrealistic assumptions or otherwise contradicts prior or background knowledge. As an example we use a theoretical constraint called *tractability*, but others may be considered (more later). Tractability is the constraint that a theory *f* of a real-world capacity (e.g., foraging) must be realizable by the type of system under study given minimal assumptions on its resource limitations. Tractability is useful for illustrating the theoretical cycle because it is a fundamental computational constraint, is insensitive to the type of system implementing the computation, and applies at all levels of organization (given basic background assumptions: e.g., computation takes time, its speed is limited by an upper bound). Tractability is a property of *f* that can be assessed independently of algorithmic- and implementational-level details (Frixione, 2001; van Rooij, 2008; van Rooij et al., 2019): For example, an organism could solve the foraging problem by deciding on an ordering before travel (planning) or it could compute a solution implicitly as it arises from local decisions made while traveling (the same applies to sorting in the figure shown in Box 1). An assessment of the tractability or intractability of *f* is independent of this “how” of the computation.

In relation to tractability, the foraging *f* (as stated) turns out a priori implausible. If an animal had the capacity *f* (as stated), then it would have a capacity for computing problems known to be intractable: The foraging *f* is equivalent to the known intractable (NP-hard) traveling-salesperson problem (Garey & Johnson, 1979). This problem is so hard, even to approximate (Ausiello et al., 1999; Orponen & Heikki, 1987), that all algorithms solving it require time that grows exponentially in the number of sites (*n*). For all but very small *n* values such a computation is infeasible.

The intractability of an *f* does not necessarily mean that the computational-level theory is wholly misconceived, but it does signal it is underconstrained (van Rooij, 2015). Tractability can be achieved by introducing constraints on the input and/or output domains of *f*. For instance, one could assume that the animal’s foraging capacity is limited to a small number of sites (e.g., *n* ≤ 5) or that the animal has the general capacity for a larger number of sites, but only if the amount of food per site meets some minimum criterion (e.g., *g*(*s*) ≥ *max*(*c*(*s,s*′)) + $\frac{max(c(s,s\prime ))}{n}$ for all *s* in *S*).^10^ In both cases, the foraging *f* is tractable.^11^ Theoretical considerations (e.g., tractability) can constrain the theory so as to rule out its most unrealistic versions, effectively endowing it with greater a priori verisimilitude. Moreover, theoretical considerations can yield new empirical consequences, such as predictions about the conditions under which performance breaks down (i.e., *n* > 5 vs. *g*(*s*) ≤ *max*(*c*(*s,s*′)) + $\frac{max(c(s,s\prime ))}{n}$ ; for further examples, see Blokpoel et al., 2013; Bourgin et al., 2017), and can constrain algorithmic-level theorizing (different algorithms exploit different tractability constraints; Blokpoel, 2018; Zednik & Jäkel, 2016). Thus, the theoretical cycle can improve both theory verisimilitude and theory testability.

Tractability/intractability analyses apply widely, not just to simple examples such as the ones above. The approach has been used to assess constraints that render tractable/intractable computational accounts for various capacities relevant for psychological science that span across domains and levels (Table 1), such as coherence-based belief updating (van Rooij et al., 2019), action understanding and theory of mind (Blokpoel et al., 2013; van de Pol et al., 2018; Zeppi & Blokpoel, 2017), analogical processing (van Rooij et al., 2008; Veale & Keane, 1997), problem-solving (Wareham, 2017; Wareham et al., 2011), decision-making (Bossaerts & Murawski, 2017; Bossaerts et al., 2019), neural-network learning (Judd, 1990), compositionality of language (Pagin, 2003; Pagin & Westerståhl, 2010), evolution, learning or development of heuristics for decision-making (Otworowska et al., 2018; Rich et al., 2019), and evolution of cognitive architectures generally (Rich et al., 2020). This existing research (for an overview, see Compendium C in van Rooij et al., 2019) shows that tractability is a widespread concern for theories of capacities relevant for psychological science and moreover that the techniques of tractability analysis can be fruitfully applied across psychological domains.

Building on other mathematical frameworks, and depending on the psychological domain of interest (Table 1) and on one’s background assumptions, computational-level theories can also be assessed for other theoretical constraints, such as computability, physical realizability, learnability, developability, evolvability, and so on. For instance, reconsider foraging. We discussed foraging above at only the cognitive level (Table 1, row 1), but one can also ask how a foraging capacity can be learned, developed, or evolved biologically and/or socially (Table 1, rows 2–5). In some cases, these theoretical constraints can again be assessed by analyses analogous to the general form of the tractability analysis illustrated above (for learnability, see, e.g., Chater et al., 2015; Clark & Lappin, 2013; Judd, 1990; for evolvability, see Kaznatcheev, 2019; Valiant, 2009; for learnability, developability, and evolvability, see Otworowksa et al., 2018; Rich et al., 2020). On the one hand, such analyses are all similar in spirit, as they assess the in-principle possibility of the existence of a computational process that yields the output states from initial states as characterized by the computational-level theory. On the other hand, they may involve additional constraints that are specific to the real-world physical implementation of the computational process under study. For instance, a learning algorithm running on the brain’s wetware needs to meet physical implementation constraints specific to neuronal processes (e.g., Lillicrap et al., 2020; Martin, 2020), evolutionary algorithms realized by Darwinian natural selection are constrained to involve biological information that can be passed on genetically across generations (Barton & Partridge, 2000), and cultural evolution is constrained to involve the social transmission of information across generations and through bottlenecks (Henrich & Boyd, 2001; Kirby, 2001; Mesoudi, 2016; Woensdregt et al., 2020). Hence, brain processes and biological and cultural evolution are all amenable to computational analyses but may have their own characteristic physical realization constraints.

**Fig. 2.**
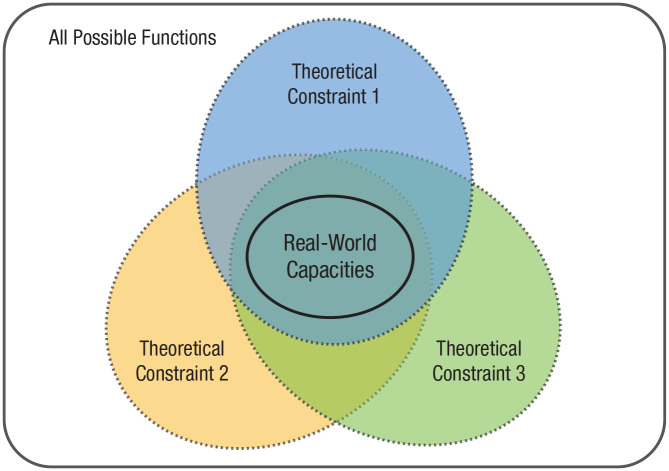
The universe of all possible functions (indicated by the rectangle) contains infinitely many possible functions. By applying several constraints jointly (e.g., tractability, learnability, evolvability) psychological scientists can reduce the subset of candidate functions to only those plausibly describing real-world capacities.

By combining different theoretical constraints one can narrow down the space of possible functions to those describing real-world capacities (Fig. 2). The theoretical cycle thus contributes to early theory validation and advances knowledge even before putting theories to an empirical test. In practice, it serves as a timely safeguard system: It allows one to detect logical or conceptual errors as soon as possible (e.g., intractability of *f*) and, in any case, before empirical tests are undertaken.

## What Effects Can Do for Theories of Capacities

We have argued that the primary aim of psychological theory is to explain capacities. But what is the role of effects in this endeavor? How are explanations of capacities (primary explananda) and explanations of effects (secondary explananda) related? Our position, in a nutshell, is that inquiry into effects should be pursued in the context of explanatory multilevel theories of capacities and in close continuity with theory development.^12^ From this perspective, the main goal of empirical research, including experimentation (e.g., testing for effects) and computational modeling or simulation, is to further narrow down the space of possible functions *after* relevant theoretical constraints have been applied. Specifically, good empirical inquiry assumes a set of a priori verisimilar theories of real-world capacities (the intersection in Fig. 2) and then proceeds to partition this set into *n* subsets. Each subset will (a fortiori) contain a high-verisimilitude theory of capacities. However, across subsets, theories may be *empirically different*: Theories in subset A may predict effects that are not predicted by theories in subset B, and vice versa. Empirical research, including testing for effects, may allow one to adjudicate among competing theories, thereby eliminating some a priori verisimilar ones that turned out to be implausible a posteriori. To the extent that theories do predict effects, and that those effects are testable experimentally, or via models or simulations, psychology is already well equipped to test those predictions. Here, we are interested in situating effects conceptually in a broader view of inquiry that also encompasses the theoretical cycle: What can effects do for theories of capacities? To answer this question, we need to accept a simple premise: that finding out that a theory is empirically inadequate is more informative if the theory is deemed verisimilar a priori than if it is already known to be implausible (e.g., the *f* intractable) before the test.

Consider again the multipliers example from Cummins (2000). Multiplication is tractable, and the partial products (M1) and successive addition (M2) algorithms meet minimal constraints of learnability and physical realizability. M1 and M2 are plausible algorithmic-level accounts of the human capacity for multiplication. But depending on which algorithm is used on a particular occasion, performance (e.g., the time it takes for one to multiply two numbers) might show either the linearity effect predicted by M2 or the step-function profile predicted by M1. Note that both M1 and M2 explain the capacity for multiplication. It is not the computational-level analysis that predicts different effects (the *f* is the same) but rather the algorithmic-level theory. In other cases, effects could follow from one’s computational-level theory (for examples from the psychology of language and logical reasoning, see Baggio et al., 2008, 2015; Geurts & van der Slik, 2005; for examples from the psychology of action, see Blokpoel et al., 2013; Bourgin et al., 2017) or from limitations of resource usage (memory or time), details of physical realization (some effects studied in neuroscience are of this kind), and so on. So one could classify effects depending on the level of analysis from which they follow. This is not a rigid taxonomy but a stepping stone for thinking about the precise links between theory and data. For example, one should want to know *why* an a priori verisimilar theory of a capacity is found to be a posteriori implausible, which is essential in deciding whether and how to repair the theory. A theory could fail empirically for many reasons, including because its algorithmic- or implementational-level analyses are incorrect (e.g., the capacity *f* is not realized as the theory says it is), because the postulated *f* predicts unattested effects or does not predict known effects despite having passed relevant tests of tractability, and so on.

Another dimension in the soft taxonomy of effects suggested by our approach pertains to the degree to which effects are relevant for understanding a psychological capacity. Some effects may well be implied by the theory at some level of analysis but may reflect more or less directly, or not at all, how a capacity is realized and exercised. For example, a brain at work may dissipate a certain amount of heat greater than the heat depleted by the same brain at rest; the chassis of an old vacuum-tube computer may vibrate when it is performing its calculations. These effects (heat or vibration) can tell us something about resource use and physical constraints in these machines, but they do not illuminate how the brain or the computer processes information. These may sound like extreme examples, but the continuum between clearly irrelevant effects such as heat or vibration and the kind of effects studied by experimental psychologists is not partitioned for us in advance: The effects collected by psychological scientists cannot just be assumed to be all equally relevant for understanding capacities across levels of analysis. We may more prudently suppose that they sit on a continuum of relevance or informativeness in relation to a capacity’s theory: Some can evince how the capacity is realized or exercised, but others are in the same relation to the target capacity as heat is to brain function.

There are no steadfast rules that dictate how or where to position specific effects on that continuum, but one may envisage some diagnostic tests. Consider again classical effects, such as the Stroop effect, the McGurk effect, primacy and recency effects, visual illusions, priming, and so on. In each case, one may ask whether the effect reveals a *constitutive aspect* of the capacity and how it is realized and exercised. Diagnostic tests may be in the form of counterfactual questions: Take effect *X* and suppose *X* was *not* the case; would that entail that the system lacks the capacity attributed to it? Would it entail that the capacity is realized or exercised differently than what the algorithmic or implementation theories hypothesize? For example, would a system not subject to visual illusions (i.e., lacking their characteristic phenomenology) also lack the human capacity for visual perception? Would a system that does not show semantic priming effects also thereby lack a capacity for accessing and retrieving stored lexical meanings? Our intent here is not to suggest specific answers but to draw attention to the fact that addressing those questions should enable us to make better informed decisions on what effects we decide to leverage to understand capacities. It also matters for whether we can expect effects to be stable across situations or tests. An effect that reveals a constitutive aspect of a capacity (one for which a counterfactual question gets an affirmative answer) may be expected to occur whenever that capacity is exercised, and so do effects that are direct manifestations of how the capacity is realized: Such effects can therefore also be expected to be replicable across experimental tests.

This brings us to a further point. Tests of effects can contribute to theories of capacities to the extent that the information they provide bears on the structure of the system, whether it is the form of the *f* it computes or the virtual machines (algorithms) or physical machines on which it is running. The contrast between qualitative (e.g., the direction of an effect) and quantitative predictions (e.g., numerical point predictions) cuts the space of effects in a way that may be useful in natural science (e.g., physics) but not in psychology. Meehl (1990, 1997) rightly criticized the use of “weak” tests for theory appraisal, but his call for “strong” tests (i.e., tests of hypotheses with risky point predictions), if pursued systematically, would entrench the existing focus in psychology on effects, albeit requiring that effects be quantitative. The path to progress in psychological science lies not in a transition from weak qualitative to strong quantitative tests but rather in strong tests of qualitative structure, that is, tests for effects that directly tap into the workings of a system as it is exercising the capacity of interest. Computational-level theories of capacities are not quantitative but qualitative models: They reveal the internal formal structure of a system, or the *invariants* that allow it to exercise a particular capacity across situations, conditions, and so on (Simon, 1990). There is usually some flexibility resulting from free parameters, but, as we have argued, principled constraints on those parameters (tractability, etc.) may be established via analyses in the theoretical cycle and by explorations of qualitative structure (Navarro, 2019; Pitt et al., 2006; Roberts & Pashler, 2000).

## Conclusion

Several recent proposals have emphasized the need to potentiate or improve theoretical practices in psychology (Cooper & Guest, 2014; Fried, 2020; Guest & Martin, 2021; Muthukrishna & Henrich, 2019; Smaldino, 2019; Szollosi & Donkin, 2021), whereas others have focused on clarifying the complex relationship between theory and data in scientific inference (Devezer et al., 2019, 2020; Kellen, 2019; MacEachern & Van Zandt, 2019; Navarro, 2019; Szollosi et al., 2020). Our proposal fits within this landscape but aims at making a distinctive contribution through the idea of the theoretical cycle: Before theories are even put to an empirical test, they can be assessed for their plausibility on formal, computational grounds; this requires that there is something to assess formally; that is, it requires a computational-level analysis of the capacity of interest. In a theoretical cycle, one addresses questions iteratively concerning, for example, the tractability, learnability, physical realizability, and so on, of the capacity as formalized in the theory. However, the theoretical cycle and the empirical cycle will need to always be interlaced: Abduced theories can then both be explanatory and meet plausibility constraints (i.e., have minimal verisimilitude) on testing; conversely, relevant effects can be leveraged to understand capacities better. The net result is that empirical tests are informative and can play a direct role in further improving our theories.

## Transparency

*Action Editors:* Travis Proulx and Richard Morey

*Advisory Editor:* Richard Lucas

*Editor:* Laura A. King

## Notes

1. Cummins (2000) uses the McGurk effect to make the same point. We mention the Stroop effect because it is among the least contested effects in psychology and easily replicable in a live in-class demonstration. Yet our point is that even uncontested, highly replicable effects are not primary explananda in psychology.

2. This is not to say that in practice this never happens (Newell, 1973). But we believe *good* theoretical practice has a different aim and starting position, as we explain later.

3. One can reasonably debate whether these capacities “carve up” the mind in the right way; and indeed this is a topic of dispute between, for example, cognitivists and enactivists (van Dijk et al., 2008). Still, few if any cognitive psychologists would maintain that the primary explananda are laboratory effects, instead of cognitive capacities, regardless of how one carves up the latter. We see the carving up as part of the activity of theory development.

4. We thank Ivan Toni for this analogy.

5. See Meehl (1997) on the “crud factor” (Lykken, 1991): In complex systems often “everything correlates with almost everything else” (p. 393); so the precise *null* hypothesis for statistical effects is seldom if ever true, but most effects may not provide much information about the key principles of operation of a complex mechanism; many will be by-effects of idiosyncratic conditions.

6. How vast is the space of possible effects? We can in principle define an unlimited number of conditions and compare them to each other. If a “condition” is some combination of values for situational variables, even if we assume only binary values (e.g., yes vs. no, presence vs. absence, greater than vs. less than), then there are 2^k^ distinct conditions that we can, in principle, define. For the number of situational variables (from low-level properties of the world, such as lighting conditions, to higher-order properties, such as the day of the week), *k* ≥ 100 is a conservative lower bound. Then there are at least 2^100^ × (2^100^ − 1) > 10^59^ distinct comparisons we can make to test for an “effect” of condition, such as the number of seconds since the birth of the universe (< 10^18^) and the world population (< 10^10^). Sampling this vast space to discover effects without any guidance of substantive theory, we are likely to “discover” many meaningless effects (“crud factor”) and fail to discover actually informative effects.

7. Even then, coding the input-output mapping as a look-up table is not explanatory, even if it is descriptive and possibly predictive (within limits). As, for instance, Cummins (2000) notes, the tide tables predict the tides well but do not explain them. One could make a list of (input, output) pairs ({1, 2}, 2), ({3, 4}, 12), ({12, 3}, 36)), but that is hardly an explanation. Moreover, the list does not allow predictions beyond the observed domain: Unless one hypothesizes that the capacity one is observing is “multiplication,” one would not be able to know the value of *x* in ({112, 3.5}, *x*). This is all the more pressing because any observations we would make in a laboratory task setting are typically a very small subset of all possible capacity inputs and outputs, and the functions that we are trying to abduce are much more complex than multiplication (e.g., compositionality; Baroni, 2020; Martin & Baggio, 2020).

8. The idea is not fully hypothetical (see Lihoreau et al., 2012), but details here are for illustration only.

9. The theory admits different orderings as long as they satisfy the output property (the constraint given by the inequality “≥”). Formally, functions are always one-to-one or many-to-one, so strictly speaking we are dealing here with a *relation*, or a *computational problem*. This is fine for characterizing capacities, which usually involve abilities to produce outputs that are invariant with respect to some property (“laws of qualitative structure”; Newell & Simon, 1976; Simon, 1990). So too in our foraging example: Depending on input details, there may be two or more routes of travel that meet the constraint; then producing at least one of them would be exercising the foraging capacity as defined above.

10. These constraints can be seen as hypothesized “normal conditions” for the proper exercise of a capacity. An intuitive argument for the *g*(*s*) ≥ *max*(*c*(*s,s*′)) + *max*(*c*(*s,s*′))/*n* constraint would be as follows: If the amount of food collected at each site exceeds *max*(*c*(*s,s*′)), the animal always collects more food than it expends energy for traveling from *s*^0^ to the *n* sites; to have enough food to cover traveling back from site *s*^n^ to *s*^0^, it needs additionally *max*(*c*(*s,s*′))/*n* at each site.

11. There may be other constraints that can achieve the same result; we invite interested readers to explore this as an exercise (for guidance, see van Rooij et al., 2019).

12. This is implicit in our view of interactions between the theoretical and empirical cycles and has been emphasized in recent philosophy of science: For example, van Fraassen (2010) discusses “the joint evolution of experimental practice and theory,” arguing that “experimentation is the continuation of theory construction by other means” (pp. 111–112).
